# Heterogeneously catalyzed transesterification reaction using waste snail shell for biodiesel production

**DOI:** 10.1016/j.heliyon.2023.e17094

**Published:** 2023-06-08

**Authors:** Alaa K. Mohammed, Zahraa A. Alkhafaje, Israa M. Rashid

**Affiliations:** Biochemical Engineering Department, Al-Khwarizmi College of Engineering, University of Baghdad, Baghdad 47024, Iraq

**Keywords:** Transesterification reaction, Biodiesel production, Waste cooking oil, Green catalyst, Central composite methodology

## Abstract

Biodiesel as an attractive energy source; a low-cost and green synthesis technique was utilized for biodiesel preparation via waste cooking oil methanolysis using waste snail shell derived catalyst. The present work aimed to investigate the production of biodiesel fuel from waste materials. The catalyst was greenly synthesized from waste snail shells throughout a calcination process at different calcination time of 2–4 h and temperature of 750–950 °C. The catalyst samples were characterized using X-Ray Diffraction (XRD), Brunauer-Emmett-Teller (BET), Energy Dispersive X-ray (EDX), and Fourier Transform Infrared (FT-IR). The reaction variables varying in the range of 10:1–30:1 M ratio of MeOH: oil, 3–11 wt% catalyst loading, 50–70 °C reaction temperature, and 2–6 h reaction time. The designed model optimization was set its parameters at 21.5 methanol molar ratio, 9.8 wt% catalyst loading, 4.8 h reaction time, and 62.2 °C reaction temperature, resulting in a mixture comprised of 95% esters content.

## 1 Introduction

Biodiesel as a sustainable alternative energy source has received the attention and concerns for investigation and utilization in existing diesel engines [1]. The manufacturing of biodiesel is generally accomplished by transesterification of triglycerides with short-chain alcohols in the presence of a suitable catalyst [2,3]. Therefore, the production of biodiesel is restricted by both feedstock and catalyst availability [4]. All vegetable oils and animal fats can be involved in the transesterification reaction but most of these refined vegetable oils are essential food chain constituents and their use for biodiesel synthesize is not preferred for competition with human food in addition to their high cost as they are refined oils [5,6]. Consequently, non-edible oils or waste vegetable oils can successfully replace the high cost refined edible oils, waste frying oil appear as a premium feedstock for biodiesel preparation [7]. The catalyzed transesterification reaction is usually accomplished using an alkaline catalyst, while the acid-catalyzed transesterification is not favorable due to long time and lower conversion [[8], [9], [10]]. Many different types of alkaline catalysts are presented to mediate transesterification; homogeneous, or heterogeneous catalysts may be employed [11,12]. Each one has some merits and drawbacks to be considered in industrial production, started with the availability, activity, to the purification steps, and reusability access [13]. In general, the more desirable catalysts are heterogeneous alkaline, since they are widely available, separable, and easily recyclable [14]. Calcium oxide (CaO) shows good catalytic activity over other metal oxides in the production of biodiesel [15]. It can be derived from many different raw materials found in nature, such as waste eggshell, waste snail shell, waste seashell, animal bones, limestone, and many other sources based on calcium carbonate (CaCO_3_) can be involved in the production of biodiesel [1].

In a study of transesterification of palm oil, a proposed conversion of 95% was achieved at 9:1 MeOH: oil molar ratio, 65 °C reaction temperature, 3 h time of reaction, and 10 wt% CaO heterogeneous catalyst prepared from mussel shell [16]. Another study was accomplished to transesterify waste cooking oil using waste chicken bones, a conversion of 89.33% was achieved, after 4 h, 65 °C, 15:1 MeOH: oil molar ratio, and 5 wt% catalyst loading [17], while Laskar et al. (2018) [19] prepared CaO catalyst from waste snail shell for esters production from soybean oil and achieve a conversion of 98% at the optimized conditions of 6:1 MeOH: oil molar ratio, 28 °C, 7 h, and 3 wt% catalyst loading. Whereas Moradi et al. (2015) [21] investigated the transesterification reaction of waste cooking oil at the optimized conditions of 22.5:1 MeOH: oil molar ratio, 75 °C, 8 h, and 9.08 wt% of solid catalyst loading prepared from demineralized water treatment precipitates. All these studies were carried out using one factor at a time optimization method, while in current study will investigate central composite design methodology to perform the superiority of current technique.

Alkhafaje et al. [9] conducted a study to produce biodiesel using waste cooking oils with a high acid number. They, as a first step, reduced the acid number of the oil to acceptable lower value, without using catalyst but instead, using high molar ratios (ethanol: oil) in esterification reaction.

In a place where snails are used as a source of food, excessive quantities of depleted shells are emitted as waste which in turn resulted in a disturbed environment, therefore, the utilize of waste shells could develop large-scale production of biodiesel in such regions [[18], [19], [20]]. This topic stated the production of a sustainable alternative energy source from food wastes that could partially resolve the environmental problems, therefore, the present work aimed to investigate the potential of waste snail shells to catalyze the methanolysis of waste cooking oil (of high acid value (4.05 mg KOH/g oil)) targeted to synthesize biodiesel product.

## 2 Materials and methods

### 2.1 Materials

The chemicals employed in this study were ethanol (Chem-Lab NV) 99.8%, methanol (Chem-Lab NV) 99.8%, KOH (Thomas Baker) 85%, phenolphthalein indicator, and methyl heptadecanoate (Sigma-Aldrich) 99.9%. Whereas waste cooking oil (WCO) and waste snail shells were collected locally.

### 2.2 Procedure

#### 2.2.1 Preparation and analysis of oil

The samples of WCO were collected from local restaurants that are specifically waste sunflower oil (the most abundant oil locally). Initially, the samples were purified from all waste insoluble particles via a filter paper (150 mm Ø), then dried at 120 °C for 6 h (in an oven from PRODIT s. a.s. Via asti 59–10026, Italy) to remove water content. The WCO was identified by acid value of 4.05 mg KOH/g oil, the high acidity content was decreased to 0.95 mgKOH/g oil following the noncatalytic esterification procedure described in our previous work [9]. The collected WCO was analyzed for fatty acids composition by the technique of gas chromatography-mass spectrometry (GC-MS) Shimadzu GC Mass 2010 QP Plus equipped with DN-WAX Capillary Column (30 m length, 0.25 mm ID, and 1 μm film thickness). The analysis was accomplished following EN 14103 standards aided with the internal standard (IS) (methyl heptadecanoate C_17:0_). The oven capillary column was kept initially at 60 °C for 2 min and raised to 200 °C at a rate of 10 °C/min holding for 1 min. Subsequently, the temperature was continued to increase from 200 °C to 240 °C at 5 °C/min rate and hold for 10 min. The detector and injector temperatures were set at 280, 260 °C, respectively with 1 μl injection volume. The analyzed WCO was comprised of 78.85% linoleic acid, 10% oleic acid, 9.42% palmitic acid, and small amounts of docosapentaenoic, eicosenoic, and docosadienoic acids. The waste oil molecular weight was estimated using Eqs. (1), (2)), where M_wav._ is the average fatty acids mixture molecular weight, f_i_ is the fatty acid mass fraction obtained from the analysis of GC-MS, M_wi_ is the molecular weight of single fatty acid, and M_woil_ is the estimated molecular weight of WCO (Budhwani et al., 2019). The waste oil molecular weight was found to be 873.9 g/mol.

(1) $${Mw}_{av.}=\frac{\sum f_{i}}{\sum \left(\frac{f_{i}}{{Mw}_{i}}\right)}$$

(2) $${Mw}_{oil}=3.{Mw}_{av.}+38.049$$

#### 2.2.2 Catalyst preparation and characterization

The waste snails were initially cleaned by washing with tap water several times, then boiled with distilled water for 1 h to remove all impurities of sand and organics. After that, it was dried for 6 h in an oven at 110 °C. The clean and completely dry snail shells were next crushed and pulverized in a grinder, then sieved at 180 μm mesh sieve and calcined under static air in a muffle furnace from 2 to 4 h calcination time, and 750–950 °C temperature range. After the calcination process, the prepared samples were stored immediately inside desiccator avoiding humidity and CO_2_ interaction from the environment.

The prepared samples were characterized using Brunauer-Emmett-Teller (BET), Fourier Transform Infrared (FTIR) Spectroscopy, X-Ray Diffraction (XRD), and Energy Dispersive X-ray (EDX) spectrophotometer analysis. BET surface area analyzer (BET: HORIBA, SA-900 series, USA) was utilized to accomplish the surface area analysis. The catalyst sample is initially going into a degassing step under vacuum and fixed temperature to eliminate any physiosorbed volatiles and impurities acquired from the atmosphere. Then an inert gas (N_2_ gas) at 77 K passes through the catalyst solid surface in a volumetric flow procedure, under a particular pressure the gas particles adsorbed on the unspecified shape of the catalyst surface forming monomolecular layer. The gas molecules will spend a finite time on the surface; and the volume of adsorbed gas is correlated with the given pressure allows to calculate the surface area of the catalyst particles. XRD measurements were conducted by X-Ray diffractometer (Shimadzu XRD 6000. Japan). Cu radiation target over a continuous Scan from Theta-2 with scan speed of 5.0000 (deg/min) and preset time 0.60 s. The functional groups attributed to the catalyst high activity were recognized using FTIR Spectroscopy device (IR Affinity-1 Shimadzu) in the range of 400–4000 cm^−1^. EDX spectrophotometer was used to identify the elemental composition of the snail shell catalyst sample.

#### 2.2.3 Transesterification of WCO

The potential of prepared heterogeneous catalyst samples has been experimented in a transesterification reaction of WCO that was conducted by mixing 20 g of WCO with a designed amount of methanol and catalyst. The catalyst going on an activation step with methanol for 40 min stirring at 40 °C prior to oil addition [[22], [23]]. A rounded bottle 3-neck flask of 500 ml volume was used to carry out the reaction. Its three necks were connected to a reflex water-cooling condenser, thermocouple, and an overhead mixing agitator to provide the reaction an adequate mixing (the mixer was set at a constant mixing speed of 450 rpm), while the desired reaction temperature was maintained with the aid of heating mantle [1]. After the transesterification reaction was completed, the mixture was moved to a separating funnel and left to settle overnight. Three layers were formed; the upper two layers were mixture of Methanol and FAME. The bottom layer was catalyst. The catalyst was filtrated from the solution mixture, washed with n-hexane several time, and dried in oven at 60C for 1 h. Finally, the catalyst was calcined at 600C for 2 h. The activated catalyst was reused and it was noted that it continued giving FAME yield of over 90% even after being used three times.

#### 2.2.4 Transesterification experimental design

Four variables at five levels were experimentally designed using central composite design (CCD) and studied to investigate the impact of reaction factors and their interactions at the heterogeneously catalyzed transesterification reaction of WCO with methanol [24,25]. Statistica program (StatSoft USA 10, Inc.) experimental design software was used to design, analyze, and optimize the affecting factors, as well as obtaining an empirical model representing the transesterification process using heterogeneous catalyst.

Table 1 presents the independent factors with their coded and actual levels selected for process optimization. The molar ratio of MeOH: oil (X_1_), catalyst loading (X_2_), reaction time (X_3_), and reaction temperature (X_4_) were used to optimize the dependent factor of reaction response fatty acid methyl ester % (FAME %) (X_5_). The polynomial model in quadratic form is represented by Eq. (3) that is suggested based on analysis of variance (ANOVA) in coded factor terms, where; b_0_, b_1_, b_2_, …b_n_ are constants [1].

(3) $$X_{5}=b_{0}+b_{1}X_{1}+b_{2}X_{2}+\ldots +b_{n}X_{n}+\sum b_{ik}X_{i}X_{k}+\sum b_{ii}X_{i}^{2}$$

**Table 1:** Independent parameters domain of transesterification reaction at the actual and coded levels.

Independent factors	Symbol	Unit	Values of each variable				
			Coded value				
			-α	−1	0	1	α
MeOH molar ratio	X_1_	mol/mol	(10	15	20	25	30)
Catalyst loading	X_2_	wt %	(3	5	7	9	11)
Reaction time	X_3_	h	(2	3	4	5	6)
Reaction temperature	X_4_	°C	(50	55	60	65	70)

### 2.3 Fatty acid methyl esters product layer analysis

The upper layer (FAME) is separated off, dried from remaining methanol in an oven at 80 °C, then weighted and stored in a dark bottle for GC analysis (GC-2014 analysis unit, Shimadzu, equipped with capillary column (DB-WAX, 30 m length, 0.25 μm film thickness, and 0.25 mm ID). The GC analysis was accomplished following EN 14103 standard that is used to estimate chromatographically the methyl ester content (wt %) of biodiesel product originally obtained from vegetable oils that do not contain C_17:0_ in its composition (all vegetable oils). The oven capillary column was kept initially at 60 °C for 2 min and raised to 200 °C at a rate of 10 °C/min holding for 1 min. Subsequently, the temperature was continued to increase from 200 °C to 240 °C at 5 °C/min rate and hold for 10 min. The detector and injector temperatures were set at 280, 260 °C, respectively. The sample preparation was accomplished by approximately mixing 250 mg of biodiesel sample with 5 ml of methyl heptadecanoate in a 10 ml vial, then 1 μl of the solution was injected for analysis. After GC analysis finished, Equation (4) was employed to calculate methyl esters content (wt%) of the biodiesel product layer [7,26].

(4) $$Esters\%=\frac{\sum_{A}-A_{IS}}{A_{IS}}.\frac{\left(C_{IS}.V_{IS}\right)}{m}*100$$

Where V_IS_: IS solution volume (ml), ∑_A_: total methyl esters peak area from C_14_ to C_24:1_, A_IS_: IS peak area, C_IS_: IS solution concentration (mg/ml), and m: biodiesel prepared sample mass (mg).

In order to evaluate the quality of biodiesel properties of the product, some properties were determined using the American Society for Testing and Materials standard (ASTM). ASTM identifies the parameters that should fulfilled before being used as a pure fuel or blended with diesel fuel [27]. The summarized results in Table 2 show that all of the measured values were in the range of test limit.

**Table 2:** Standard limit and physical property of biodiesel according to ASTM D6751.

Physical property	Test method	Standard limit	Values obtained from current study	Reference
Specific gravity (15 °C) g/cm^3^	ASTM D-941	0.86 to 0.9	0.86	[28]
Flash Point ^°^ C	ASTM D-93	Min 93	120	[29]
Pour Point ^°^ C	ASTM D-92	−15 to +10	0	[29]
Acid Number mg KOH/g of oil	ASTM D-664	Max 0.5	0.1	[28]

### 2.4 Data, value and validation

#### 2.4.1 Catalyst characterization

**Fig. 1:**
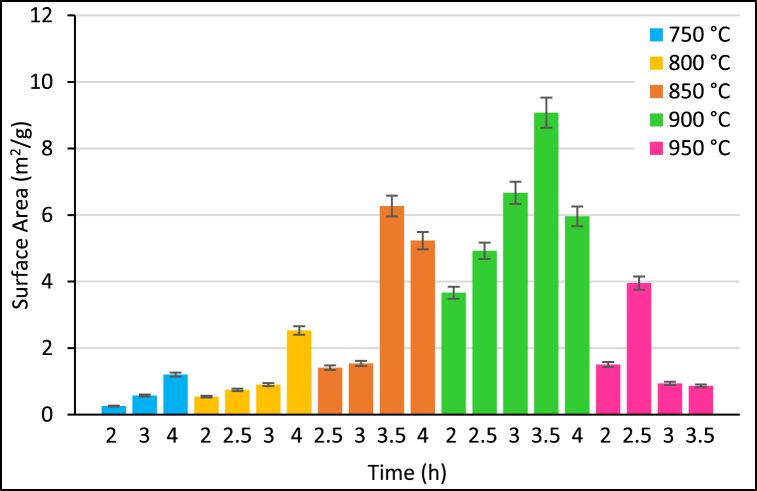
BET surface area analysis results for various calcination conditions of time and temperature of the prepared CaO catalyst.

The catalyst activity which is identified by its adsorption/desorption properties is enhanced at the high specific surface area [4]. Therefore, the effect of calcination conditions was initially investigated for the best surface area, the temperature of calcination and time are significantly manipulating specific surface area and active sites of the prepared CaO catalyst as described in Fig. 1. The specific surface area was found to be increased with the increase in calcination conditions due to the modification of sample composition during calcination. Gaseous CO_2_ elimination at high calcination temperatures of 750, 800, 850, 900, and 950 °C causes the formation of pores at the catalyst surface [16]. Progressive increase in the surface area was observed at a calcination temperature of 750, 800, 850 °C for various calcination time without reaching the effective surface area. This is probably due to the decomposition of CaCO_3_ into CaO is not complete yet, while the higher surface area (9.29 m^2^/g) was formed at 900 °C, 3.5 h when CaCO_3_ was completely converted into CaO. However, prolonged heating did not result in higher surface area and lead to agglomeration in which the particles aggregate together and ending in sintered powder as it was observed at 950 °C. The obtained result is close to Laskar et al. (2018) [19] who obtained a close BET result (7 m^2^/g) of calcined snail shells at their optimized calcination conditions of 900 °C, 4 h. This variation in results may be attributed to the source of raw CaCO_3_ used and different calcination conditions.

**Fig. 2:**
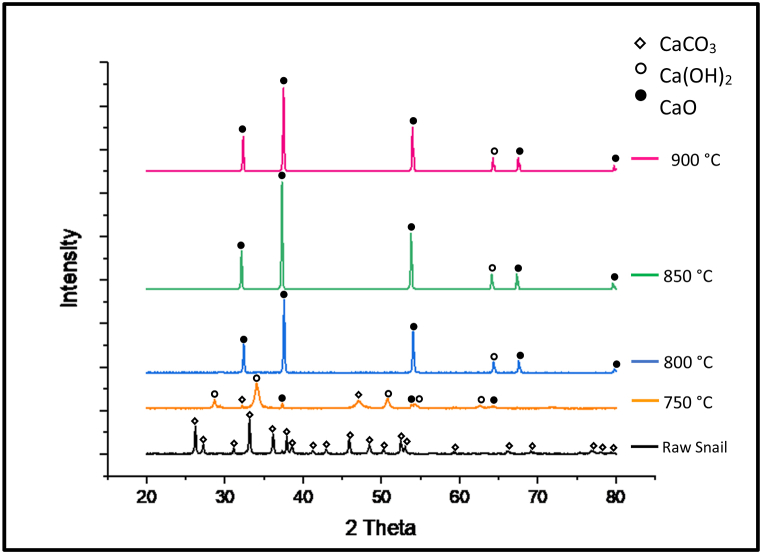
XRD patterns of raw and calcined snail shell powder at 3.5 h calcination time for calcination temperatures of 750, 800, 850, and 900 °C.

The raw and calcined snail shell XRD patterns are presented in Fig. 2. It can be observed that the uncalcined snail shell revealed the crystalline nature of the catalyst and was dominated by the aragonite crystalline CaCO_3_ phase. The intense peaks that belong to CaCO_3_ can be observed at the strongest peaks of 2θ = 27.26°, 33.18°, 36.19°, 37.93°, and 45.899°. CaCO_3_ was subsequently converted into CaO with an increase in calcination temperature, and show the CaO patterns identification at 800, 850, and 900 °C, while 750 °C was not enough to produce a highly active CaO catalyst. This interesting result proves the transform of CaCO_3_ into CaO at high temperatures. The most modifying calcination temperature in snail shell samples was 900 °C. This probably due to the elimination of gaseous CO_2_ molecules that was not accomplished yet, some CO_2_ molecules still adhere to the catalyst surface at a calcination temperature of 750, 800, and 850 °C while completely eliminated at 900 °C, which was the end of CaCO3 decomposition. The sharp and intense peaks of the calcined snail shell evidence the crystallinity structure of CaO catalyst. The obtained results are well matched with Laskar et al. (2018) [19] who reported similar XRD patterns for uncalcined and calcined snail shells. Boro et al. (2014) [[30], [31]] also stated similar XRD patterns when calcined waste shells. Whereas, Nur Syazwani et al. (2015) [32] found that all calcium carbonite was completely transformed into CaO during the calcination process of angel wing shells based on XRD analysis.

**Fig. 3:**
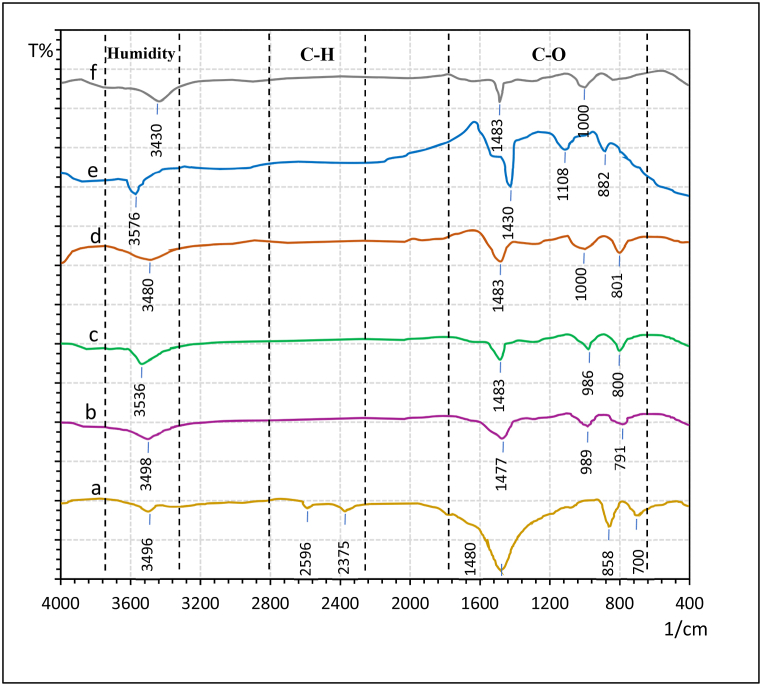
FTIR results for (a) raw snail shell, as well as calcined snail shell at 3.5 h for (b) 750 °C, (c) 800 °C, (d) 850 °C, (e) 900 °C, and (f) 950 °C.

The raw and calcined snail shell powders were recorded with FTIR spectra to identify the absorption bands of presented functional groups. Fig. 3 shows the FTIR patterns of the raw and calcined snail shell at different calcination temperatures. For raw snail shell in Fig. 3 (a), the asymmetric molecules stretching of CO_3_^−2^ is related to the major absorption band at 1480 cm-1, while the observed absorption bands at 700 and 858 cm-1 are attributed to the in-plane and out-plane vibration modes band for CO_3_^−2^ molecules, these peaks are attached to the existence of CaCO_3_ in raw snails powder [33]. Whereas, the obtained peaks at 2375 and 2596 cm^−1^ are attributed to the organic matters presented in the shells [30], which completely disappeared after the calcination temperature of 750 °C. The shift in the absorption bands that are ascribed with CO_3_^−2^ to high energy in Fig. 3 (b, c, d, e, and f) is attributed to the loss of carbonate ion and decrease in the mass of functional groups attached to CO_3_^−2^ ions that conformed the decomposition of CaCO_3_ to CaO throughout the calcination progress [1]. The presence of broad peaks at 3400–3600 attributed to the formation of Ca(OH)_2_ in the calcined samples and presence of humidity in the raw shells, this result demonstrates the tendency of highly active calcined snail shells to react with moisture content from the air and CO_2_ molecules [34]. The obtained infrared spectra results agreed well with the other research works [1,19,32].

**Fig. 4:**
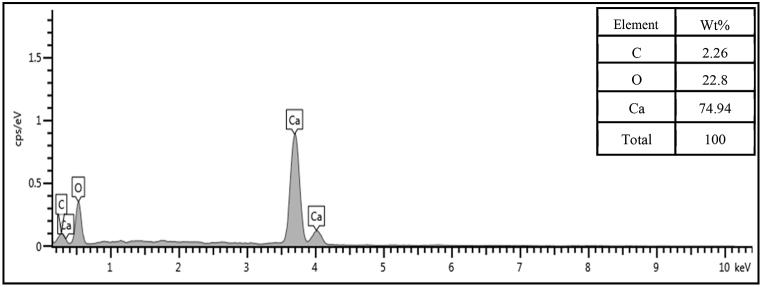
EDX elemental analysis of the prepared CaO catalyst at 900 °C and 3.5 h calcination conditions.

Furthermore, the quantitative elemental composition analyzed by EDX confirmed the presence of calcium essential element at the appreciable quantity (74.9 wt%) (Fig. 4), therefore, the snail shell is a worthy raw material in the catalyzed biodiesel production. Eventually, the snail shell derived catalyst shows its best catalytic activity at 900 °C and 3.5 h of calcination conditions based on the previously established characterization results.

Viriya et al. [35] studied the basicity of the snail shell catalyst using CO2-TPD analysis. They showed that the catalyst possessed two major functions of medium (54 μmol g ^−1^ at 400–550 °C) and strong (133 μmol g ^−1^ at temperature >550 °C) base sites. The experimental results suggested that the basic amount of the strongly basic sites strongly corresponded to the activity of the catalysts; namely, the higher basic mount brought about the higher biodiesel yield.

#### 2.4.2 Design of experiments using CCD

The obtained experimental values of FAME content were compared with the predicted values estimated using CCD that is illustrated in Table 3. The thirty design experiments ending in a close deviation of the predicted and actual response, that resulted in a coefficient of determination R_2_ of 0.936 and adjusted R_2_ of 0.905 in a difference of 0.03 between R_2_ and adj R_2_ that is lower the allowable difference of 0.2 [1], approves the model accuracy, the closer R_2_ value to unity the model will be more accurate and gives predicted values closer to the actual response.

**Table 3:** Transesterification reaction experimental design results following CCD.

run	MeOH molar ratio (X_1_)	Cat. loading (wt%) (X_2_)	Reaction time (h) (X_3_)	Reaction temp. (°C) (X_4_)	FAME% (wt%) (experimental)	FAME% (wt%) (predicted)	Residuals
1	15	5	3	55	57.750	56.848	0.902
2	25	5	3	55	62.458	62.256	0.202
3	15	9	3	55	66.124	64.548	1.576
4	25	9	3	55	70.983	70.062	0.921
5	15	5	5	55	74.932	73.900	1.032
6	25	5	5	55	77.750	77.805	−0.055
7	15	9	5	55	80.416	79.685	0.731
8	25	9	5	55	82.924	83.695	−0.771
9	15	5	3	65	78.924	74.777	4.147
10	25	5	3	65	81.083	78.290	2.793
11	15	9	3	65	85.052	80.895	4.157
12	25	9	3	65	86.858	84.514	2.344
13	15	5	5	65	88.416	85.813	2.603
14	25	5	5	65	89.624	87.823	1.801
15	15	9	5	65	93.192	90.017	3.175
16	25	9	5	65	94.753	92.132	2.621
17	10	7	4	60	70.991	76.413	−5.422
18	30	7	4	60	82.458	83.936	−1.478
19	20	3	4	60	76.625	79.887	−3.262
20	20	11	4	60	88.258	91.896	−3.638
21	20	7	2	60	59.958	64.740	−4.782
22	20	7	6	60	87.291	89.409	−2.118
23	20	7	4	50	55.625	54.443	1.182
24	20	7	4	70	72.725	80.807	−8.082
25	20	7	4	60	90.332	89.412	0.920
26	20	7	4	60	88.834	89.412	−0.578
27	20	7	4	60	89.572	89.412	0.160
28	20	7	4	60	89.362	89.412	−0.050
29	20	7	4	60	88.622	89.412	−0.790
30	20	7	4	60	89.752	89.412	0.340

The model empirical equation terms that are significantly validated is clearly presented in Eq. (5) that is representing the heterogeneously catalyzed transesterification reaction using waste snail shell derived catalyst.

(5) $$X_{5}=-1037.6955+5.4907\;X_{1}+7.8589\;X_{2}+52.0691\;X_{3}+29.3220\;X_{4}-0.0924\;{X_{1}}^{2}-3.0845\;{X_{3}}^{2}-0.2179\;{X_{4}}^{2}$$

## 3 ANOVA results

The analysis of variance displayed in Table 4 records ANOVA evaluations. These results imply the effective parameters in the regression model by estimating its *P*-Value, it becomes significant when the *P*-Value is less than 0.05. It can be observed that the most significant factor was the reaction temperature in its linear form (X_4_), followed by the time of reaction (X_3_), and subsequently by the quadratic reaction temperature term (X_4_^2^).

**Table 4:** Statistical ANOVA results of transesterification reaction parameters findings.

terms	SS	df	Mean square	F-value	*P*-value	Comments
X_1_	84.893	1	84.893	5.58162	0.032085	significant
X_1_^2^	146.293	1	146.293	9.61857	0.007297	significant
X_2_	216.324	1	216.324	14.22303	0.001848	significant
X_2_^2^	21.251	1	21.251	1.39721	0.255592	
X_3_	912.889	1	912.889	60.02123	0.000001	significant
X_3_^2^	260.952	1	260.952	17.15727	0.000869	significant
X_4_	1042.643	1	1042.643	68.55244	0.000001	significant
X_4_^2^	813.751	1	813.751	53.50304	0.000003	significant
X_1_.X_2_	0.011	1	0.011	0.00072	0.978976	
X_1_.X_3_	2.261	1	2.261	0.14863	0.705264	
X_1_.X_4_	3.593	1	3.593	0.23623	0.633966	
X_2_.X_3_	3.669	1	3.669	0.24124	0.630420	
X_2_.X_4_	2.501	1	2.501	0.16445	0.690820	
X_3_.X_4_	36.186	1	36.186	2.37920	0.143791	
Error	228.141	15	15.209			
Total SS	3542.781	29				

### 3.1 Transesterification process optimization using CCD

The model accuracy was confirmed by transesterification variables optimization. The attained experimental value confirmed the validity of the model as shown in Table 5, the result was holding 0.499% error between the actual value obtained experimentally and the predicted data suggested by design software, the optimal FAME % was then set as 95.1%.

**Table 5:** Transesterification reaction optimization following designed model.

Process parameters (coded)	X_1_	X_2_	X_3_	X_4_	Predicted FAME %	Actual FAME %	Error %
Actual parameters	MeOH molar ratio	Catalyst loading (wt %)	Reaction time (h)	Reaction temp. (°C)			
Optimal values	21.5	9.8	4.8	62.2	95.605	95.106	0.499

### 3.2 Transesterification parameters interactions

#### 3.2.1 Effect of reaction temperature and time

**Fig. 5:**
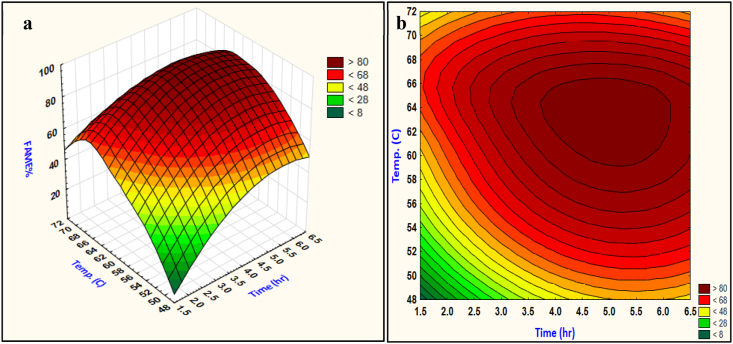
FAME% 3D response as a function of reaction temperature and time (a) surface response, (b) contour plot.

The graphical results, shown in Fig. 5 (a) and (b), present the 3D response surface and contours of the interactive effect of temperature and time. As supposed, the reaction conversion increases as the reaction temperature and time increase. Obviously can be observed from Fig. 5 (a), the temperature is the most manipulating factor on transesterification, an increase in the reaction temperature resulted in high reaction conversion because the reaction molecules will be supplied with more energy, but a prolonged reaction temperature is not favorable. Temperature in the range of (60–66) °C is successive for reaction completion, while higher temperatures have a negative impact on transesterification, as the methanol molecules going to evaporate from the reaction mixture and resulted in an unstable molar ratio.

The effect of the reaction time is also of considerable effect as shown in Fig. 5 (b). An increase in the methyl esters production is obtained at balanced conditions of time and temperature. The reaction time between 3.5 and 5.5 h is adequate, extended time do not help in increasing the conversion because the reaction equilibrium is reached, temporarily minimize the range of temperature and time do not result in enhancing the biodiesel production.

The interactive reaction time and temperature are effective. From Fig. 5 (b) at a time less than 3.5 h the temperature is hardly affecting the reaction for high conversion, meanwhile, when the temperature is far from methanol boiling point the reaction resulted in unfavorable conversion for all time range since the reaction requires its’ enough time and appropriate temperature for completion.

The obtained results are well agreed with the literary works; the temperature was treated as an important variable to enhance biodiesel production [36,37].

#### 3.2.2 Effect of molar ratio and catalyst loading

**Fig. 6:**
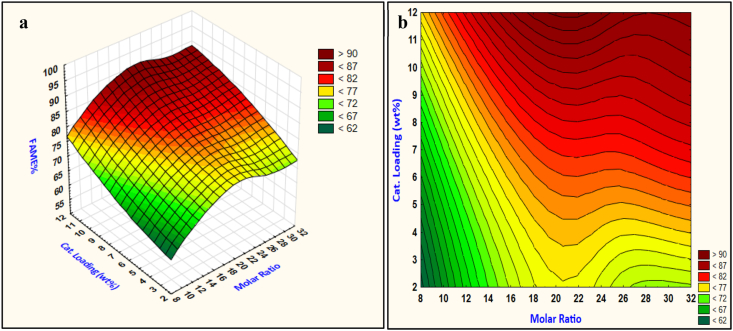
Reaction response of interactive molar ratio and catalyst loading (a) surface response and (b) contour plot.

The effect of interactive molar ratio and catalyst loading is shown in Fig. 6 (a) and (b) below. The 3D surface response in Fig. 6 (a) indicated that the methyl esters production increases when increasing the methanol quantities as well as catalyst loading.

Excess methanol stoichiometry is used to shift the reaction toward completion, higher reaction conversion is promoted when a high alcohol ratio is employed, but excessive amounts of methanol make glycerol recovery very difficult and resulted in a dispersed glycerol layer with esters layer. Furthermore, a higher methanol molar ratio resulted in a dilution effect and cause the product of methyl esters to hold a large amount of alcohol which is unfavorable in the purification and recovery of the products [37]. Therefore, alcohol molar ratio is a fundamental variable in transesterification to be studied and optimized for certain production parameters of time, temperature, and catalyst concentration [37].

The catalyst loading, on the other hand, is strongly affecting biodiesel production. Since applying a high catalyst amount causes the reaction conversion to be increased but excessive addition resulted in the slurry formation and decreases the production of methyl esters as the viscosity of the mixture increases and leads to reduced diffusion between the reagents [1].

**Fig. 7:**
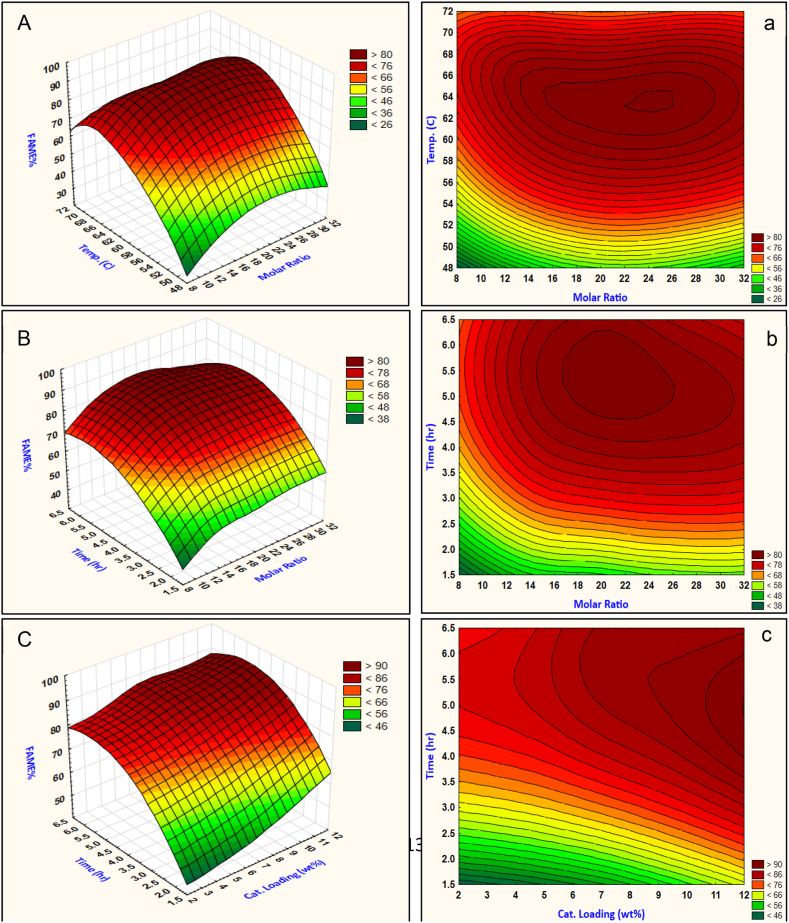
Transesterification parameters Interactions, (A, a) Interaction of molar ratio and temperature, (B, b) Interaction of molar ratio and time, (C, c) Interaction of catalyst loading and time, (D, d) Interaction of catalyst loading and temperature. Upper case for surface plots, and lower case for contour plots.

The interactive response of molar ratio and catalyst loading resulted in an interesting effect, the response shows that the molar ratio between 20 and 22 and catalyst loading in the range of 9–11 wt% resulted in maximum biodiesel production. As can be noticed from Fig. 6 (b) the reaction conversion cannot be at a high level at less than 5.5 wt% catalyst loading and high molar ratio do not affect the reaction high conversion at these levels, otherwise, when the methanol molar ratio is less than 10 the catalyst loading has no change on the reaction conversion for high methyl esters production. Buasri et al. (2013) [16] recognized an effective methanol molar ratio of 22.5–24, while Moradi et al. (2015) [21] found a range of catalyst loading between 9 and 12 wt% that is well agreed with the established values of methanol molar ratio and catalyst loading. The remaining interactions are all shown in Fig. 7(a–d).

## 4 Conclusion

The heterogeneously catalyzed transesterification reaction was statistically modeled and experimentally conducted using central composite design methodology. The catalytic activity of prepared CaO snail shell derived catalyst was investigated and exhibited an excellent catalytic activity in transesterification reaction at the optimized calcination conditions of 900 °C and 3.5 h. The synthesized catalyst was characterized by 9.29 m^2^/g specific surface area. Accordingly, FAME product was obtained at high purity of 95% at the optimized reaction conditions of the molar ratio of 21.5:1 MeOH: oil, 9.8 wt % catalyst loading, 4.8 h reaction time, and 62.2 °C reaction temperature. The ANOVA study in transesterification reaction shows that the reaction temperature has the most significant effect on the reaction, followed by reaction time and catalyst loading whereas, the molar ratio has the less significant effect from other reaction parameters in the studied range of transesterification conditions. The temperature is constrained by the boiling point of the alcohol since transesterification is adversely affected by high temperatures. This is due to the fact that the alcohol molecules will evaporate from the reaction mixture, creating an unstable molar ratio.

The low-grade biodiesel feedstocks specifically WCO and waste snail shell, were very adequate for biodiesel synthesis. The low-grade feedstock employment could reduce the production cost, drop the struggle between food and fuel engineering, and develop a sustainable technology throughout recycling the wastes into a useful biodiesel product, and finally aid to improve citizens' awareness about the significance of waste recycling.

## Sources of financing format

No special funding was provided for this study by the government, businesses, or nonprofit organizations.

## Author contribution statement

Alaa K. Mohammed: Conceived and designed the experiments.

Israa M. Rashid: Analyzed and interpreted the data; Contributed reagents, materials, analysis tools or data; Wrote the paper.

Zahraa A. Alkhafaje: Performed the experiments; Wrote the paper.

## Data availability

Data will be made available on request.

## Declaration of competing interest

The authors declare that they have no known competing financial interests or personal relationships that could have appeared to influence the work reported in this paper.
